# New chemometrics-assisted spectrophotometric methods for simultaneous determination of co-formulated drugs montelukast, rupatadine, and desloratadine in their different dosage combinations

**DOI:** 10.1186/s13065-024-01345-6

**Published:** 2024-11-19

**Authors:** Marco M. Z. Sharkawi, Nehal F. Farid, Moataz H. Hassan, Said A. Hassan

**Affiliations:** 1 Pharmaceutical Analytical Chemistry Department, Faculty of Pharmacy, Beni-Suef University, Alshaheed Shehata Ahmad Hegazy St, Beni-Suef, 62514 Egypt; 2 Pharmaceutical Analytical Chemistry Department, College of Pharmaceutical Sciences and Drug Manufacturing, Misr University for Science and Technology, 6th of October City, 12566 Giza Egypt; 3 Pharmaceutical Analytical Chemistry Department, Faculty of Pharmacy, Cairo University, Kasr El-Aini Street, Cairo, 11562 Egypt

**Keywords:** Artificial neural network, Desloratadine, Genetic Algorithm, Montelukast, Rupatadine, Partial least squares

## Abstract

Two accurate, precise and robust multivariate chemometric methods were developed for the simultaneous determination of montelukast sodium (MON), rupatadine fumarate (RUP) and desloratadine (DES). These methods provide a cost-effective alternative to chromatographic techniques by utilizing spectrophotometry in pharmaceutical quality control. The proposed approaches, partial least squares-1 (PLS-1) and artificial neural network (ANN), were optimized using genetic algorithm (GA) to select the most influential wavelengths, enhancing model performance. A five-level, three-factor design was employed to construct a calibration set with 25 mixtures, utilizing concentration ranges of 3–19, 5–25, and 4–20 µg.mL^−1^ for MON, RUP, and DES, respectively. An independent validation set was employed to assess the performance of the models. GA significantly improved the PLS-1 and ANN models for RUP and DES, though minimal enhancement was observed for MON. These methods were successfully applied to the simultaneous quantification of the compounds in pharmaceutical formulations and proved useful as stability-indicating assays for RUP, given that DES is a known degradation product. The developed methods offer a valuable tool for impurity profiling and quality control in pharmaceutical analysis.

## Introduction

**Fig. 1:**
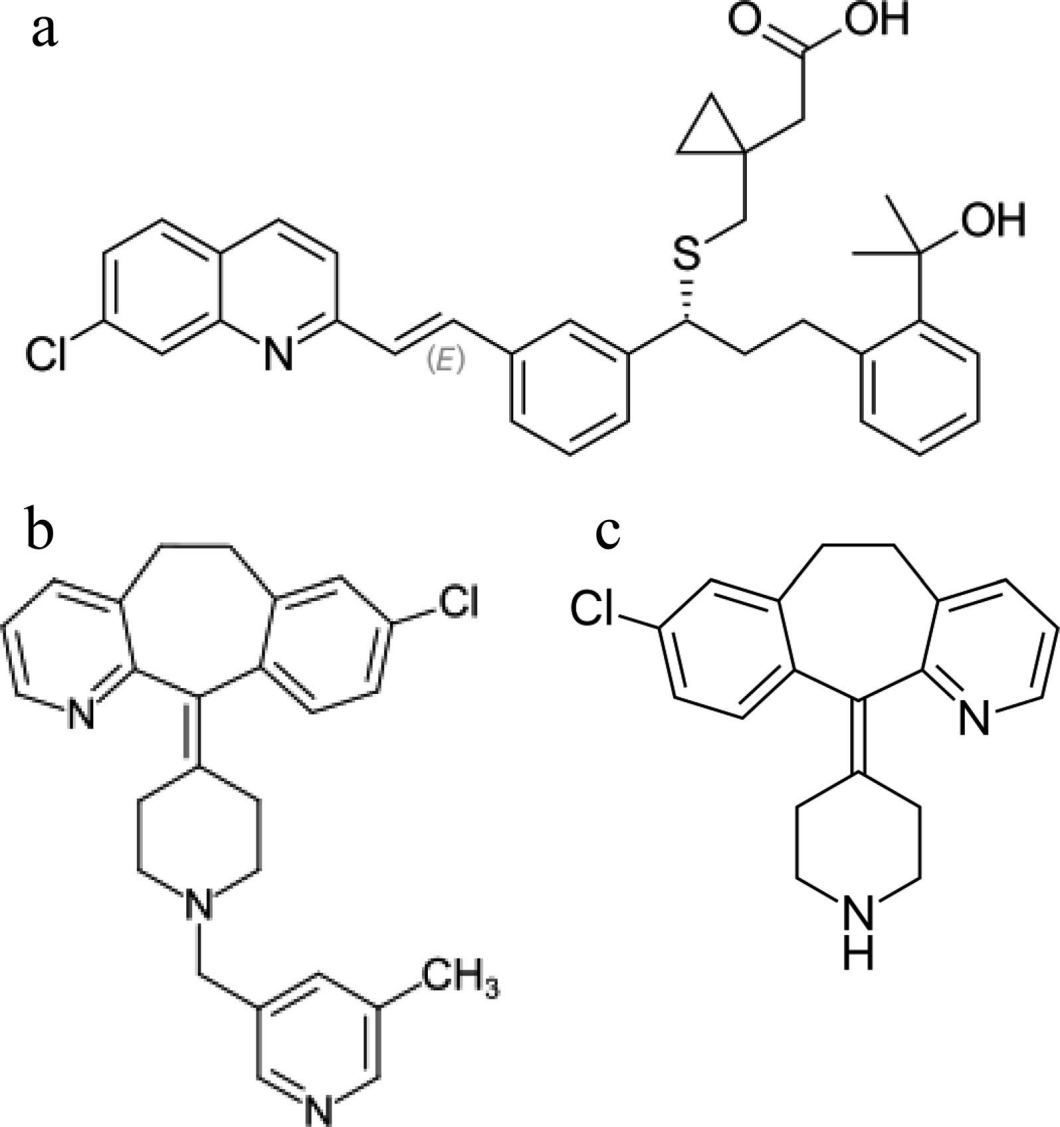
Structural formulae for (a) MON, **b** RUP, and **c** DES

Montelukast sodium (MON), Fig. 1a, is a selective leukotriene receptor antagonist and has been used as a potent therapeutic agent in the treatment of chronic asthma [1]. Rupatadine fumarate (RUP), Fig. 1b, is a second generation antihistaminic with platelet activating factor antagonist activity [2], that is used as a therapeutic agent for the treatment of chronic urticarial, allergic rhinitis and conjunctivitis [3]. Desloratadine (DES), Fig. 1c, is a potent antihistaminic that is known to be one of the main active metabolites of RUP [4]. DES is reported to be impurity B of RUP in BP [5], and it is described as a degradation product of RUP [6]. MON and RUP are co-formulated as film coated tablets e.g. Smarti M® and Rupanex M^®^, while MON and DES are combined in tablets called Desolid-M^®^. MON, RUP, and DES are official in British Pharmacopeia (BP) [5].

Literature review revealed several reports for determination of MON [7–9], RUP [10, 11], and DES [12–14]. Both MON and RUP were determined simultaneously by various techniques such as derivative spectrophotometry [15], spectrofluorimetry [16], HPTLC [17] and HPLC [18–22]. MON and DES were determined by spectrofluorimetry [23] and HPLC [24, 25]. RUP and DES were determined by HPLC [6, 26]. To the best of our knowledge, there is only one HPLC method that have been reported for the simultaneous determination of the three drugs under investigation [27].

Pharmaceutical product quality has been critical to patient safety. Presence of degradation products or impurities can alter physicochemical, pharmacological and toxicological properties of the drugs, and hence they influence both safety and efficacy of pharmaceutical products [28]. Therefore, pharmaceutical organizations and health authorities increased their standards regarding drugs’ impurities and degradation products. ICH guideline Q3A(R2) classified impurities in new drug substances into organic, inorganic and residual solvents impurities [29]. FDA requires forced degradation studies to be included for new drugs registrations [30]. So, performing stability studies became a main demand in pharmaceutical industries to ensure product quality throughout the shelf lifetime, and to ensure absence of degradation products in pharmaceutical products.

Several analytical techniques are used for stability indicating and impurity testing with chromatographic methods dominating this field [31–34]. However, the prevalence of spectrophotometers, their simple operation and their lower operation cost can introduce spectrophotometric methods as a powerful alternative to chromatographic ones. The spectral overlap between drugs and their degradation products and impurities represents a challenge, due to their structural similarity. Various spectral manipulations present potentials for solving spectral overlap arising from such structural similarity [35–39].

Multivariate regression and design of experiment methods have various applications in analytical chemistry [40–45]. When combined with spectrophotometric methods, multivariate regression methods are a powerful tool for processing spectral data. They have been successfully applied for the determination of drugs in the presence of their impurities and/or degradation products [46–48]. Chemometric methods employ various regression algorithms such as multivariate curve resolution-alternating least squares (MCR-ALS) and partial least squares (PLS) and machine learning techniques, e.g. artificial neural networks (ANN) and support vector machines (SVM). In PLS, spectral data matrix is decomposed via principal component analysis into scores and loading matrices, where the model is built based on the optimum number of latent variables (LVs) [49]. ANN is a machine learning algorithm that mimics human nervous system in its building blocks (neurons), and uses various learning methods to build the model [50]. Genetic algorithm (GA) is a variable selection algorithm that imitates the biological evolution mechanisms to attain the most substantial data [51]. It proved success in improving the prediction power and reducing dimensionality of the data [52–54].

This work aims to introduce two chemometric models, namely partial least squares-1 (PLS-1) and artificial neural network (ANN), for determination of the ternary mixture of MON, RUP, and DES in their different dosage form combinations. The models will also act as a stability indicating method for RUP determination, as DES is known to be its degradation product and is impurity B according to BP. The mixture was used for comparing the two models and to study the effect of genetic algorithm (GA) variable selection on their performance. Therefore, four models were built for quantitation of these drugs, namely PLS-1, GA-PLS, ANN, and GA-ANN.

## Experimental

### Instruments and software

The absorbance measurements were done on Shimadzu dual-beam UV–visible spectrophotometer (Kyoto, Japan), model UV-1650 using 1-cm quartz cells. UV-Probe software version 2.21 (Shimadzu) was used to automatically obtain the spectral data. Multivariate methods were applied using MATLAB^®^ 9.2.0.538062 (R2017a). PLS was carried out by using PLS toolbox 2.1 (Eigenvector Research, Inc., Manson, USA), while ANN and GA were performed using MATLAB toolboxes.

### Materials and reagents

Montelukast sodium and desloratadine were generously supplied by Global Napi Co. (Giza, Egypt). Rupatadine fumarate was kindly supplied by Mash premiere (Cairo, Egypt). Their purity was assessed according to their official methods in BP [5] and was found to be 99.85% ± 0.89 for MON, 99.35% ± 0.55 for DES, and 99.62% ± 0.75 for RUP. Smarti-M^®^ tablets manufactured and distributed by Zydus Healthcare limited (Ahmedabad, India) labelled to contain 10.375 mg montelukast sodium and 12.79 mg rupatadine fumarate. Analytical grade methanol was purchased from Piochem Co. (Giza, Egypt).

### Standard solutions

Stock standard solutions of the three drugs (1 mg.mL^−1^) were prepared in methanol. Working standard solutions for each drug (100 µg.mL^−1^) were prepared by suitable dilutions from stock solutions in methanol. All solutions were freshly prepared and wrapped in aluminum foils.

### Procedures

#### Construction of calibration set and model development

A five-level, three-factor design was employed for the preparation of the calibration mixtures [55]. The design included 25 mixtures representing the calibration set, with different concentrations of the examined drugs. The drug concentrations were coded from −2 to + 2, with center levels of 11, 15, and 12 µg.mL^−1^ for MON, RUP, and DES, respectively (Table 1). To prepare the required mixtures, precise aliquots were transferred from the working standard solutions of the three drugs into a series of 10-mL volumetric flasks, then diluted with methanol to achieve the desired concentrations. Spectral data utilized were in the range of 221–400 nm for MON, and 221–300 nm for both RUP and DES, both data ranges with 1.0 nm intervals. The data were transferred to MATLAB for further manipulation. Models were then generated for PLS-1 and ANN, and their optimization was investigated through the implementation of GA as a variable selection tool.

**Table 1:** The concentrations of MON, RUP, and DES in different mixtures used in the calibration and validation sets

Mixture no	MON (µg.mL^−1^)	RUP (µg.mL^−1^)	DES (µg.mL^−1^)
1	11	15	12
2	11	5	4
3	3	5	20
4	3	25	8
5	19	10	20
6	7	25	12
7	19	15	8
8	11	10	8
9	7	10	16
10	7	20	20
11	15	25	16
12	19	20	12
13	15	15	20
14	11	25	20
15	19	25	4
16	19	5	16
17	3	20	4
18	15	5	12
19	3	15	16
20	11	20	16
21	15	20	8
22	15	10	4
23	7	5	8
24	3	10	12
25	7	15	4
26*	10	10	10
27*	11	15	12
28*	5	25	5
29*	18	5	18
30*	3	20	18
31*	19	6	6
32*	18	22	4
33*	6	6	20
34*	15	15	0
35*	16	0	8

^*^Mixtures of validation set

#### Construction of validation set and assessment of model performance

Ten mixtures containing different ratios of the three drugs, within the ranges used in the calibration set, were prepared as an independent validation set to test and compare the predictive abilities of the designed models (Table 1). The criteria used to assess the model performance included recovery percentage (R%), root mean square error of prediction (RMSEP) and the linearity parameters of the plot between predicted and actual concentrations. Additionally, root mean square error of calibration (RMSEC), and root mean squares error of cross validation (RMSECV) calculated during model development, were used for evaluation.

#### Application of the proposed multivariate chemometric methods for the determination of MON and RUP in Smarti-M^®^ tablets

Ten tablets were accurately weighed and finely powdered. An accurately weighed amount of the powder equivalent to 10 mg of MON and RUP were transferred to a 100-mL volumetric flask, and 30 mL methanol were added, then the solution was sonicated for 30 min. After sonication, the solution was filtered into a 100-mL volumetric flask, and the volume was completed to the mark with methanol. Different aliquots were transferred from the prepared solution into a series of 10-mL volumetric flasks and their volumes were completed to the mark using methanol. The UV spectra of the prepared solutions were recorded, and their data were transferred to MATLAB, then the concentrations of the cited drugs were predicted by the proposed models.

## Results and discussion

Chemometrics is one of the most effective methods that reflects the power of using spectrophotometric methods augmented by statistical and mathematical tools. This work focuses mainly on the simultaneous determination of a ternary mixture of MON, RUP and DES in their different dosage forms. Additionally, introducing spectrophotometers as a powerful alternative to the more common chromatographic instruments.

**Fig. 2:**
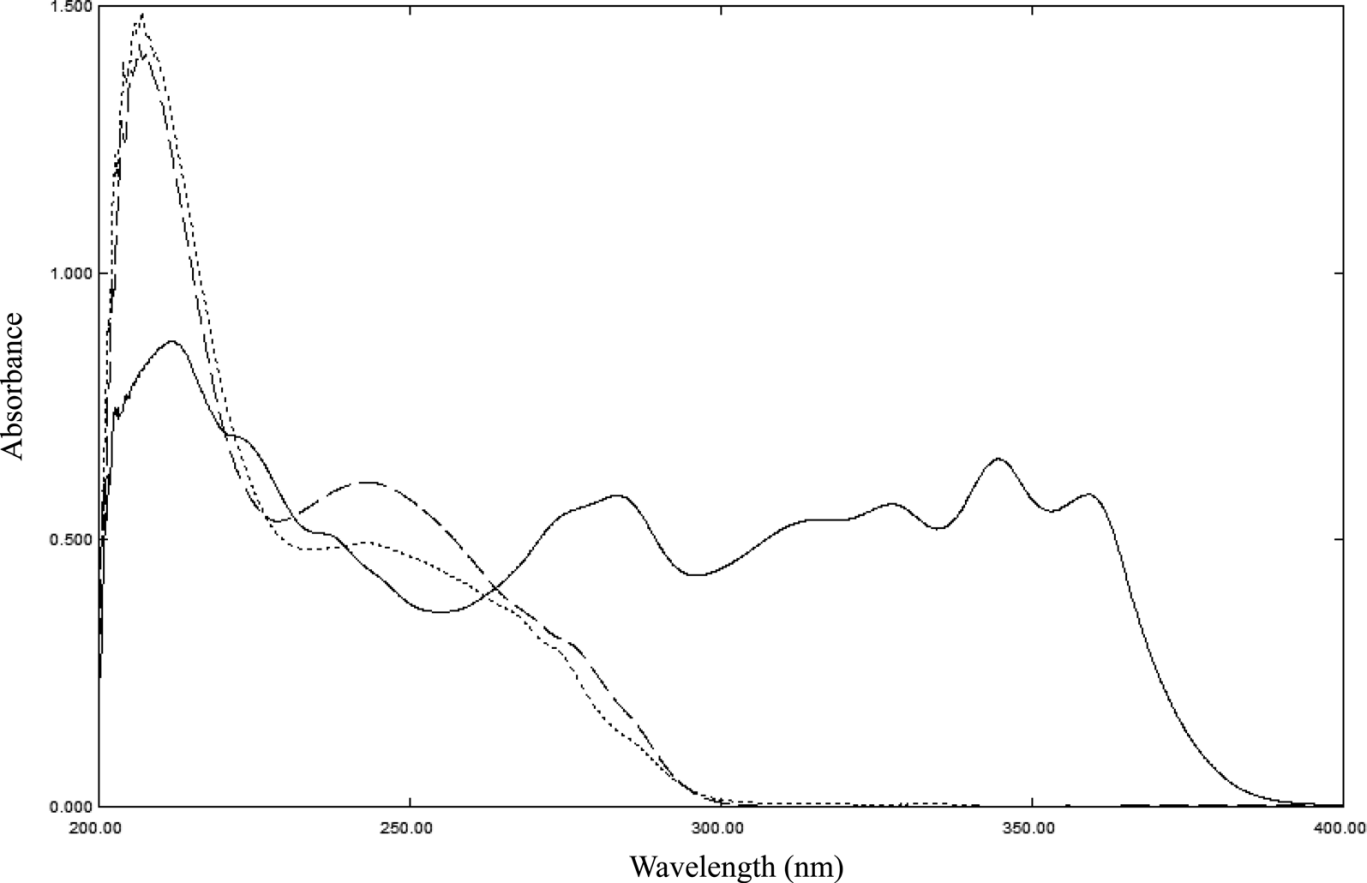
Zero order absorption spectra of 15 µg/mL MON (─), RUP (....), and DES (─) using methanol as blank

As shown in Fig. 2**,** determination of the three compounds using conventional spectrophotometric approaches is challenging due to the spectral overlap in the range 200–300 nm. Although MON can be easily determined in the range of 300–400 nm, the spectra of RUP and DES are severely overlapped which hinder their direct determination. Being a known impurity and degradation product to RUP [5, 6] and one of its main metabolites [4], DES chemical structure is closely related to that of RUP (Fig. 1) explaining their spectral overlap. Consequently, resolving their spectra by traditional univariate spectrophotometric methods is not feasible. Therefore, multivariate models were chosen as a fast, accurate and selective alternative to solve such a problem.

### Calibration and validation sets

The calibration set was designed using five-level three-factor approach in the concentration ranges of 3–19 µg.mL^−1^, 5–25 µg.mL^−1^ and 4–20 µg.mL^−1^ with center levels of 11, 15, and 12 µg.mL^−1^ for MON, RUP and DES, respectively. An independent validation set was established to test the predictive power of the proposed models. The set consisted of 10 mixtures containing different ratios of the three drugs, with some of them are only binary mixtures to simulate the commercial dosage forms in the market that either contain MON-RUP or MON-DES (Table 1**)**. The five concentration levels and linearity ranges for the three compounds were chosen based on their spectral signals at the selected wavelengths, so that the absorbance of the mixtures in calibration and validation sets does not exceed the linearity.

The data ranges were selected for MON models in the range of 221–400 nm, while for RUP and DES data points were in the range of 221–300 nm. Data points below 221 nm were excluded due to high absorbance of the compounds in this region which exceeded the limit of the spectrophotometer and represent noise to the proposed models. The data in range 301–400 nm was excluded for RUP and DES, as they do not show absorbance in this range.

### Partial least squares (PLS-1 models)

**Fig. 3:**
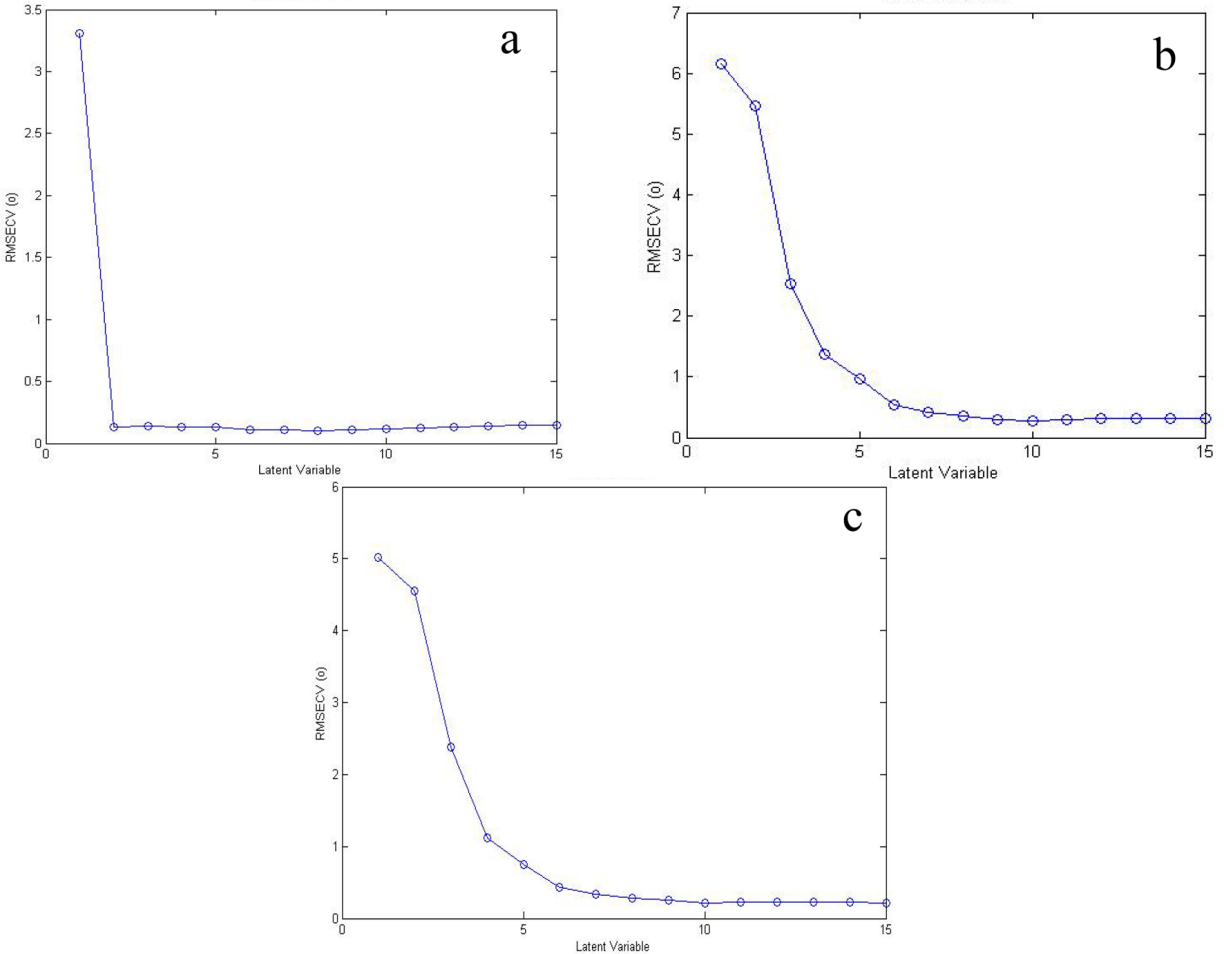
The plot of RMSECV versus LVs of (**a**) MON, **b** RUP, and **c** DES showing optimum LVs for PLS-1 models

This model is used for factor analysis and is constructed for exclusively each compound. It is based on a single concentration vector of the target compound in the calibration set, which is the key distinction between PLS-1 and PLS-2. Consequently, PLS-1 has the advantage of optimizing the model specifically for each compound rather than collectively for the three compounds. The optimal number of latent variables (LVs) is important to avoid the problem of model overfitting [56]. The number of LVs was determined using the leave-one-out cross-validation approach, with the ideal number of LVs for each compound being the one with the lowest RMSECV. Two LVs were found to be ideal for MON, while six LVs were optimal for both RUP and DES, as shown in Fig. 3. The disparity in the number of LVs between MON on one hand and RUP and DES on the other hand, suggests that MON is easier to quantify due to its extended spectrum compared to the other two drugs, whose similar and overlapping spectra required six LVs for resolution. The PLS-1 models were successfully applied to predict the concentrations of the three compounds in all validation set mixtures, as shown in Table 2. The high predictive accuracy of the proposed PLS-1 model was further confirmed by excellent calibration parameters, as presented in Table 3.

**Table 2:** Determination of MON, RUP, and DES in the validation set by the proposed models

Mix. No	Concentration (µg.mL^−1^)			Recovery %^a^											
				PLS-1			GA-PLS			ANN			GA-ANN		
	MON	RUP	DES	MON	RUP	DES	MON	RUP	DES	MON	RUP	DES	MON	RUP	DES
26	10	10	10	99.13	97.01	102.48	99.41	99.17	100.83	99.38	99.26	101.35	99.32	99.01	101.12
27	11	15	12	99.79	99.17	100.27	100.01	99.55	100.07	99.99	100.63	99.63	100.05	99.83	99.21
28	5	25	5	98.75	100.23	97.49	99.12	99.38	100.50	99.42	99.51	99.93	99.02	99.32	101.44
29	18	5	18	99.98	97**.**00	100.95	100.06	98.93	100.27	99.92	97.16	100.69	99.98	99.40	100.63
30	3	20	18	97.17	98.98	101.26	97.38	99.67	100.76	98.30	99.47	100.93	99.09	99.76	100.74
31	19	6	6	99.47	97.57	100.41	99.59	97.13	102.10	99.45	97**.**00	102.98	99.51	97.02	102.69
32	18	22	4	100.16	100.17	98.30	100.21	100.28	97.14	100.30	100.55	100.95	100.37	99.82	100.93
33	6	6	20	99.70	103**.**00	98.46	99.94	102.33	99.78	99.56	100.92	100.21	100.63	101.75	99.76
34	15	15	0	99.78	102.67	NA	99.75	100.98	NA	99.77	101.34	NA	99.77	100.52	NA
35	16	0	8	99.60	NA	97.00	99.67	NA	97.98	99.54	NA	99.26	99.81	NA	99.44
	Mean			99.35	99.53	99.62	99.51	99.71	99.93	99.56	99.54	100.66	99.75	99.60	100.66
	SD			0.87	2.23	1.88	0.82	1.44	1.51	0.53	1.56	1.10	0.53	1.26	1.09
	RMSEP^b^			0.060	0.208	0.188	0.048	0.114	0.098	0.054	0.130	0.107	0.049	0.102	0.010

^a^Average of three determinations

^b^Root mean square error of prediction

**Table 3:** Statistical parameter values for simultaneous determination of MON, RUP, and DES using the optimized chemometric methods

Parameter of interest	PLS-1			GA-PLS			ANN			GA-ANN		
	MON	RUP	DES	MON	RUP	DES	MON	RUP	DES	MON	RUP	DES
Concentration range (µg.mL^−1^)	3–19	5–25	4–20	3–19	5–25	4–20	3–19	5–25	4–20	3–19	5–25	4–20
No. of LV / hidden neurons	2	6	6	2	5	5	1	15	3	1	8	1
No. of wavelengths	180	80	80	48	26	30	180	80	80	48	26	30
RMSEC^a^	0.085	0.217	0.197	0.082	0.104	0.116	0.076	0.155	0.070	0.065	0.072	0.033
RMSEP^b^	0.060	0.208	0.188	0.048	0.114	0.098	0.054	0.130	0.107	0.049	0.102	0.010
RMSECV^c^	0.130	0.549	0.428	0.113	0.167	0.156	NA	NA	NA	NA	NA	NA
Accuracy^d^ (mean ± SD)	99.35 ± 0.87	99.53 ± 2.23	99.62 ± 1.88	99.51 ± 0.82	99.71 ± 1.44	99.93 ± 1.51	99.56 ± 0.53	99.54 ± 1.56	100.66 ± 1.1	99.75 ± 0.53	99.6 ± 1.26	100.66 ± 1.09
Precision^e^	0.88	2.24	1.89	0.82	1.44	1.51	0.53	1.57	1.09	0.53	1.27	1.08
Intercept^f^	−0.0696	−0.0955	−0.0653	−0.0534	−0.0122	−0.0370	−0.0376	−0.0728	0.0213	−0.0202	−0.0067	0.0628
Slope^f^	1.0021	1.0049	1.0054	1.0019	0.9989	1.0043	1.0003	1.0040	1.0038	1.0000	0.9972	0.9988
Correlation coefficient (r)^f^	1.0000	0.9996	0.9995	1.0000	0.9999	0.9998	0.9999	0.9998	0.9999	0.9999	0.9999	0.9999

^a^Root Mean Square Error of Calibration

^b^Root Mean Square Error of Prediction

^c^Root Mean Squares Error of Cross Validation

^d^The accuracy (n = 3), mean recovery of the concentrations in the validation set mixtures

^e^The interday precision (n = 3), RSD of the recovery of the concentrations in the validation set mixtures measured in three days

^f^Data of the straight line plotted between predicted concentrations of each component versus actual concentrations of validation set

### Genetic algorithm optimization and GA-PLS models

Genetic algorithm simulates and mimics the biological evolution and the natural selection theory, as it utilizes structures based on data that are very similar to chromosomes to encode information. GA’s main goal is to recognize an original population of solutions and then depends on survival of the fittest approach to select the best solutions [51]. It is mainly employed as a function optimization technique that improves data selection to determine the most substantial data points to obtain the best results. The development of an efficient selection model requires careful selection of the GA parameters. For optimization of these parameters, R% and RMSEP of the validation set mixtures were compared to choose the best parameters.

Firstly, several population sizes were tried ranging from 20 to 200, and it was concluded that the optimum population size for MON and RUP were 50, and for DES was 20. The number of variables per window varied from 2 to 20, and the optimum number of variables was two for each analyte. In all fittings, the mutation rate was kept at 0.005 and single breeding crossover with random cross validation were applied. The number of LVs used in GA optimization was the same used to build PLS-1 models, two for MON and six for both RUP and DES. Parameters of GA are summarized in Table 4.

**Table 4:** Parameters of the genetic algorithms

Parameter	Value
Population size	MON, RUP (50) DES (20)
Maximum generations	50
Mutation rate	0.005
The number of variables in a window (window width)	2
Percent of population the same at Convergence	100
% wavelengths used at initiation	50
Crossover type	Single
Maximum number of latent variables	MON (2) RUP, DES (6)
Cross validation	Random
Number of subsets to divide data into for cross validation	4
Number of iterations for cross validation at each generation	2

**Fig. 4:**
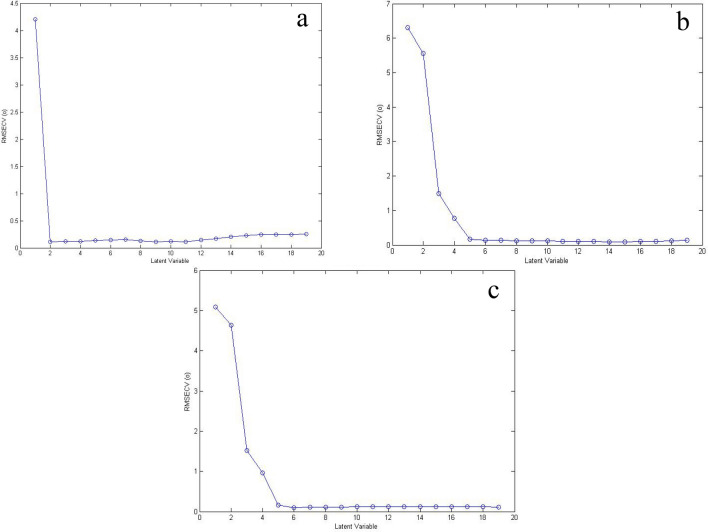
The plot of RMSECV versus LVs of (**a**) MON, **b** RUP, and **c** DES showing optimum LVs for GA-PLS models

Upon applying the optimized GA, data points number decreased from 180 to 48 for MON, from 80 to 26 for RUP, and from 80 to 30 for DES. This means that GA was able to decrease the data points number to about 27–37%. The selected data was used for building GA-PLS models for the three compounds, and the optimum number of LVs was optimized as presented in Fig. 4. The ideal number of LVs for MON was not changed from its PLS-1 model (two). This can be explained by the fact that the data of the extended part of MON spectrum (300–400 nm) were the main contributors in building the PLS-1 model and were also selected by GA as the most significant wavelengths in GA-PLS. On the contrary, the number of LVs for both RUP and DES decreased from six to five, when GA was applied as shown in Table 3. Choosing the best variables by GA may have attributed to this decrease in LVs. It is also worth mentioning that RMSEC, RMSEP, and RMSECV values were decreased for RUP and DES when GA was applied indicating better selectivity. This is in contrast to MON, whose numbers were not significantly changed between PLS-1 and GA-PLS models. GA was also able to improve the prediction of the concentrations of the three compounds in the validation set. This can be noticed in Table 2, where the values of R% and standard deviation (SD) in GA-PLS models were better than those in PLS-1 for RUP and DES.

### Artificial neural networks (ANN and GA-ANN models)

ANN operates by a network of neuron-based structures composed of three layers: input, hidden, and output. Feed forward networks were employed in the study, while back propagation was used for the learning process [57]. In ANN, data is introduced to the input layer, weights are then produced according to the inputs then converted to output values using various transfer functions. Several parameters should be taken into consideration in order to get the optimum ANN performance, as the number of hidden neurons, transfer functions, and training functions.

Various transfer functions were investigated, namely purelin-purelin, logsig –purelin and tagsig-purelin. Purelin-purelin was found to be the most suitable one, mainly due to the linear correlation between absorbance and concentration of the three compounds. Many training functions were examined for networks training, and with no difference in performance between them, the training function Levenberg–Marquardt (TRAINLM) was chosen as it is less time consuming. The validation set was integrated into the training process to limit overfitting of the models, and training was terminated when root mean square error (RMSE) of calibration set decreased while that of validation set was increased.

**Fig. 5:**
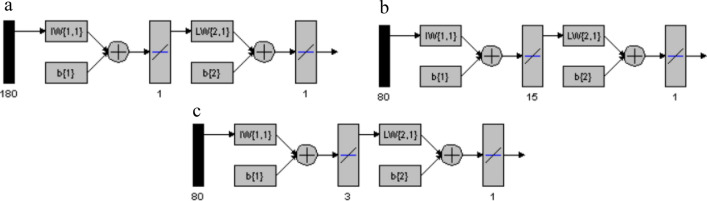
Different layers of the networks used for (**a**) MON, **b** RUP, and **c** DES prediction using ANN

The number of neurons in the hidden layer was examined for each compound, resulting in one neuron for MON, fifteen for RUP, and three for DES, as shown in Fig. 5. This can be attributed to MON's extended spectrum, where the lack of interference from other components required less processing for the MON model. In contrast, the significant overlap between the RUP and DES spectra required more neurons to handle the additional complexity. All the optimized parameters for the ANNs are summarized in Table 5. The ANN models successfully predicted the concentrations of the three compounds in all validation set mixtures as shown in Table 2. The high predictive accuracy of the proposed ANN models was further confirmed by excellent calibration parameters (Table 3**)**.

**Table 5:** Optimized parameters of ANNs

Method	ANN			GA-ANN		
Drug	MON	RUP	DES	MON	RUP	DES
Hidden neurons number	1	15	3	1	8	1
Transfer functions	Purelin–Purelin			Purelin–Purelin		
Learning coefficient	0.001			0.001		
Learning coefficient decrease	0.001			0.001		
Learning coefficient increase	100			100		

**Fig. 6:**
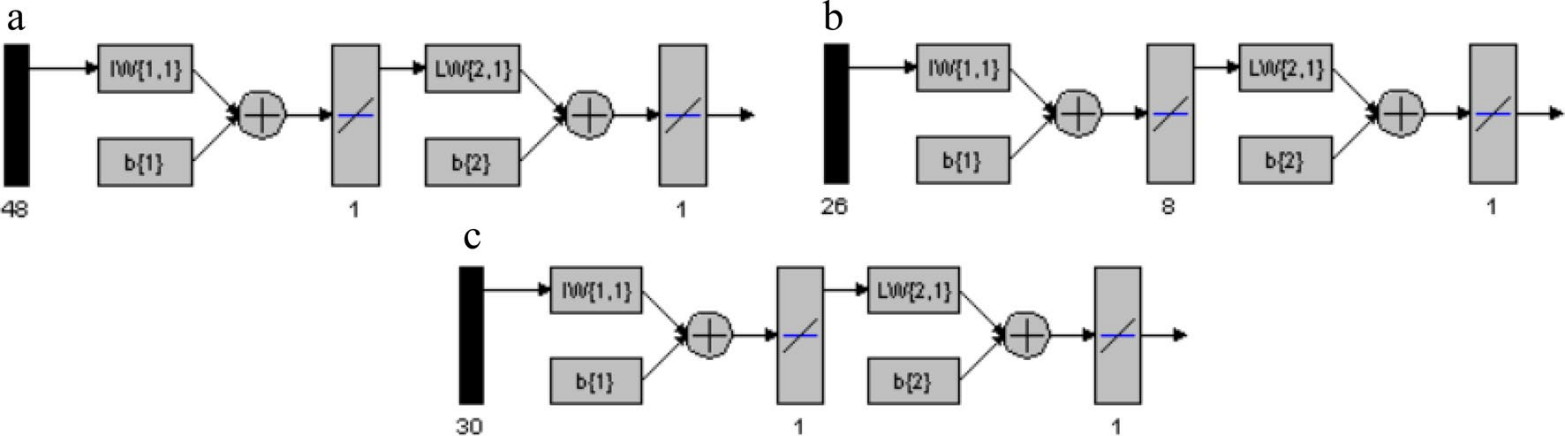
Different layers of the networks used for (**a**) MON, **b** RUP, and **c** DES prediction using GA-ANN

When ANNs were built using GA data (GA-ANN models), the neurons number for MON remained the same (one). This confirms the previous suggestion that GA data does not improve model performance for MON, due to the extended part of its spectrum which influences both GA and non-GA models equally. On the other hand, great performance improvement was obtained in GA-ANN models for RUP and DES, and the number of neurons decreased to eight and one, respectively (Fig. 6). This proves that GA was successfully able to elect the most influential variables from the data, which in turn represented a smaller number of inputs that required less neurons for processing. Both RMSEC and RMSEP were minimized for RUP and DES upon using GA data for ANN as shown in Table 3. Moreover, GA-ANN improved the prediction of all the concentration of the validation set mixtures and enhanced the R% and SD as shown in Table 2.

### Statistical parameters of the proposed models

To evaluate the performance of the models, several statistical parameters—such as error values, accuracy, precision, and linearity—were calculated. RMSEC refers to the error calculated for the 25 mixtures in the calibration set, while RMSECV represents the error calculated during leave-one-out cross-validation for the calibration set. RMSEP is the error calculated for the 10 mixtures in the validation set. These error metrics are commonly used to assess the performance of chemometric models. Although there are no specific acceptability criteria for these errors, lower values are generally preferred.

When using GA, a significant reduction in error values was observed for RUP and DES, with decreases ranging from 20 to 90%. In contrast, the reduction in error values for MON was less pronounced, ranging from 3 to 14%, as shown in Table 3. This can be explained by the fact that MON has an extended spectrum compared to the other drugs, which contributes equally to its performance in both the raw data models and the GA models.

The accuracy of the models was evaluated by calculating the recovery percentage (R%) for the validation set mixtures, with all recovery values falling within the recommended range of 100 ± 3%, as displayed in Table 3. Precision was evaluated by calculating the relative standard deviation percentage (RSD%) of the recoveries from the validation set mixtures, measured over three days. The recommended RSD% should be ≤ 2%. MON consistently showed the lowest RSD%, with values below 1% across all models. In contrast, both RUP and DES exhibited higher RSD% values in all models (Table 3). Specifically, RUP and DES had RSD% values above 1%, with the highest being 2.24% and 1.89%, respectively, in the PLS-1 models based on raw data. This can be attributed to their spectral overlap, which limited the performance of the PLS-1 model. However, applying the GA model improved these results. Calibration curves were constructed by plotting the predicted concentrations of each drug against their theoretical concentrations in the validation set. The regression parameters for these lines are summarized in Table 3. The models produced satisfactory correlation coefficient values (≥ 0.999) and slopes close to 1 for all three drugs, with intercepts being very low, as recommended.

### Application of the proposed models on dosage form

Upon the application of the proposed chemometric models to determine MON and RUP in Smarti-M® tablets, good recoveries were obtained (Table 6**)**. To compare the performance of the proposed chemometric methods and the reported method [15], Student's t-test and variance ratio F-test were applied. No significant difference was observed, indicating the good predictive ability of the proposed models as shown in Table 6.

**Table 6:** Statistical comparison for the results obtained by the proposed chemometric models and the reported method for the analysis of MON, RUP, and DES in Smarti-M® tablets

Parameters	PLS-1		GA-PLS		ANN		GA-ANN		Reported Method [15]^a^	
	MON	RUP	MON	RUP	MON	RUP	MON	RUP	MON	RUP
Mean^b^	101.28	100.63	101.12	100.79	101.13	101.26	100.99	101.32	100.85	101.34
SD	1.10	0.97	1.11	1.01	1.60	1.29	1.26	1.35	1.97	1.30
n	9	9	9	9	9	9	9	9	9	9
Variance	1.205	0.949	1.232	1.022	2.568	1.672	1.586	1.815	3.876	1.693
Student's t test^c^ (2.12)	0.575	1.326	0.37	1.015	0.336	0.142	0.181	0.032	-	-
F value^c^ (3.44)	3.217	1.784	3.147	1.657	1.509	1.012	2.444	1.072	-	-

^a^Spectrophotometric determination of MON and RUP using first derivative spectrophotometry

^b^ Average of three determination

^c^ The values in the parenthesis are the corresponding theoretical values of t and F at P = 0.05

### Greenness assessment and comparison to reported chromatographic methods

As the focus on developing environmentally sustainable analytical methods grows, it is essential to evaluate the greenness of the proposed chemometric method in comparison to reported chromatographic approaches. This assessment was conducted using two established tools: the Green Analytical Procedure Index (GAPI) [58] and the AGREE metric [59].

The GAPI tool is widely recognized for its comprehensive evaluation of the environmental impact of an analytical method, covering the entire process from sample collection to final evaluation. It uses five pentagrams with color-coded segments—green (low impact), yellow (moderate impact), and red (high impact)—to visually represent the environmental burden. The GAPI assessment of the proposed chemometric method demonstrated a clear environmental advantage over most reported chromatographic methods. Specifically, the pentagram for the proposed method (Table 7) includes seven green zones, reflecting its eco-friendly nature. The three red zones, which correspond to off-line sample collection, the use of methanol, and the absence of waste treatment, are the only areas where environmental impact is noted. Overall, the method's minimal waste generation and reduced energy consumption contribute to its high level of greenness.

**Table 7:** Comparison between the proposed chemometric method and the reported HPLC methods for analysis of MON, RUP, and DES

Parameters	Proposed method	Reported method [18]	Reported method [19]	Reported method [20]	Reported method [21]	Reported method [22]	Reported method [27]
Technique	chemometrics-assisted spectrophotometry	HPLC–PDA	HPLC–PDA	HPLC–PDA	HPLC–PDA	HPLC–UV	HPLC–UV
Analytes (linearity range, µg.mL^−1^)	MON: (3–19) RUP: (5–25) DES: (4–20)	MON and RUP (5–15)	MON and RUP (15–40)	MON and RUP (10–80)	MON: (10–50) RUP: (10–60)	MON and RUP (100–300)	MON, RUP, and DES (1–10)
Solvents and reagents used	Methanol	Methanol, acetonitrile, buffer, and phosphoric acid	Methanol, water, triethylamine, and phosphoric acid	Methanol, acetonitrile, buffer, and phosphoric acid	Acetonitrile and buffer	Acetonitrile, buffer, and phosphoric acid	Ethanol, water, triethylamine, and phosphoric acid
AGREE							
GAPI							

In contrast, the reported chromatographic methods [18, 19, 21, 22] exhibit a higher environmental impact, as indicated by their four red zones and fewer green zones (five) in their GAPI pentagrams. The red and yellow zones, located in the lower-right sections of the pentagrams (Table 7) correspond to significant waste production, higher energy consumption, and a lack of waste treatment. One reported method [20] shows a closer level of greenness to the proposed method, featuring six green zones but still exhibiting three red zones, primarily due to higher energy consumption. Another reported method [27] is comparable to the proposed method, displaying only two red zones but also fewer green zones (six versus seven). This method uses a greener solvent but consumes more energy.

For a more detailed and quantitative comparison, the AGREE metric was applied. This tool evaluates the environmental impact based on the 12 principles of Green Analytical Chemistry (SIGNIFICANCE). The output is displayed as a colored, clock-shaped pictogram, with an overall score ranging from 0 to 1 and a color gradient from green (more eco-friendly) to red (less eco-friendly). The closer the score is to 1, and the color to green, the greener the method [59].

The proposed chemometric method achieved a score of 0.68, outperforming the reported chromatographic methods, which scored between 0.56 and 0.64 (Table 7). Despite the use of methanol, which is not considered a green solvent, the proposed method demonstrated significant advantages in terms of minimal waste generation, higher sample throughput, and reduced energy consumption. These benefits are reflected in the green zones of Sects. 7, 8, and 9 of the AGREE pictogram. Conversely, the reported methods show higher environmental burdens due to their more extensive use of solvents, greater energy demands, and higher waste production.

The combined results from the GAPI and AGREE assessments clearly demonstrate the proposed method's superiority in terms of energy efficiency, lower solvent consumption, and reduced waste generation, making it a more environmentally sustainable option compared to the chromatographic methods.

In addition to its greenness, another important factor in comparing the methods is their ability to detect multiple analytes. While only one reported method [27] was capable of detecting all three analytes under investigation, the remaining methods were limited to detecting only MON and RUP. This highlights the broader applicability of the proposed chemometric method, which, like method [27], is suitable for stability-indicating assays of RUP. Moreover, the proposed models showed higher sensitivity than the reported methods [19–22] as demonstrated by the linearity ranges presented in Table 7.

In summary, the proposed chemometric method surpasses the reported chromatographic methods in terms of greenness, sensitivity, and applicability. It offers superior environmental performance, reduced energy and solvent consumption, and minimal waste generation. These advantages make the proposed method a more sustainable and eco-friendly solution for pharmaceutical quality control, particularly for stability-indicating assays and impurity profiling in pharmaceutical analysis.

## Conclusion

Advanced chemometrics-assisted spectrophotometric methods were developed for the determination of a ternary mixture of MON, RUP, and DES. Two models, namely PLS-1 and ANN, were constructed and were then optimized using genetic algorithms (GA), resulting in GA-PLS and GA-ANN models. GA was successfully employed as a variable selection tool, with its performance compared to models based on raw data. The GA-PLS and GA-ANN models for RUP and DES demonstrated enhanced performance compared to traditional PLS-1 and ANN models. However, GA models for MON did not show significant improvement, likely due to MON’s extended spectral range. The proposed models were able to determine the compounds in the validation set mixtures and dosage form. Additionally, since DES is an official impurity and a known degradation product of RUP, these models can be utilized for stability-indicating assays of RUP across various dosage forms. The models proved to be reliable, offering fast, accurate, and sensitive analysis of the cited drugs, while being cost-effective, energy-efficient, and less time- and solvent-consuming compared to the sophisticated chromatographic methods. These advantages establish the proposed models as an excellent tool to offer spectrophotometry as an alternative and reliable option for quality control units.

## Data Availability

The data analyzed during the current study are available from the corresponding author on reasonable request.

## Abbreviations

AGREE: Analytical greenness metric approach
ANN: Artificial neural network
DES: Desloratadine
GA: Genetic algorithm
GAPI: Green analytical procedure index
ICH: International council for harmonisation
LVs: Latent variables
MON: Montelukast sodium
PLS: Partial least squares regression
RMSEC: Root mean square error of calibration
RMSECV: Root mean square error of cross validation
RMSEP: Root mean square error of prediction
RSD%: Relative standard deviation percentage
RUP: Rupatadine fumarate
SD: Standard deviation
